# Sustained Effects of Glucagon-Like Peptide-1 (GLP-1) Agonists on Blood Pressure in Obesity and Type 2 Diabetes: A Longitudinal Case Study

**DOI:** 10.7759/cureus.88893

**Published:** 2025-07-28

**Authors:** Jimmy Joseph

**Affiliations:** 1 Internal Medicine, Aster DM Healthcare, Dubai, ARE

**Keywords:** diabetes, glp-1 agonist, hypertension, obesity, semaglutide

## Abstract

Glucagon-like peptide-1 receptor agonists (GLP-1 RAs) confer multiple cardiometabolic benefits beyond glycemic control, including weight reduction and potential cardiovascular protection. This case presents an 18-month follow-up of a 48-year-old obese male with type 2 diabetes mellitus (T2DM) and hypertension, who achieved sustained blood pressure (BP) control and lipid stability on semaglutide following discontinuation of triple antihypertensive therapy. At baseline, the patient had a BMI of 38 kg/m², BP of 156/96 mmHg, and an elevated glycosylated hemoglobin (HbA1c). Semaglutide was initiated at 0.25 mg weekly and titrated to 1 mg over six months. The patient exhibited progressive improvements in glycemic control, body weight, and BP, with HbA1c reduction and weight loss sustained over 18 months. Notably, BP remained consistently ≤130/80 mmHg, and lipid parameters remained stable without additional pharmacotherapy. This case underscores the potential of GLP-1 RAs to exert antihypertensive effects, possibly through weight loss, vascular endothelial function enhancement, and modulation of the renin-angiotensin-aldosterone system (RAAS). These findings support the evolving role of GLP-1 RAs as a multifaceted therapeutic option in managing T2DM with coexisting cardiovascular risk factors.

## Introduction

Glucagon-like peptide-1 receptor agonists (GLP-1 RAs) have emerged as powerful agents in the management of type 2 diabetes mellitus (T2DM), offering glycemic control with added cardiovascular and renal benefits. Their role in reducing major adverse cardiovascular events (MACE), promoting weight loss, and delaying renal progression has led to widespread incorporation in international diabetes guidelines. Notably, emerging evidence points to significant effects of GLP-1 RAs on hypertension and lipid modulation, even in the absence of overt dyslipidemia. Hypertension coexists with T2DM in over 60% of patients and amplifies the risk of macrovascular complications. Obesity, insulin resistance, and low-grade inflammation drive both diseases through overlapping mechanisms, including sympathetic overactivation, sodium retention, and vascular remodeling. Traditional antihypertensive therapies, though effective, often require multiple agents and may be poorly tolerated, emphasizing the need for complementary strategies. Early trials, such as the Liraglutide Effect and Action in Diabetes (LEAD) program, noted modest blood pressure (BP) reductions with GLP-1 RAs [1]. The Semaglutide Unabated Sustainability in Treatment of Type 2 Diabetes (SUSTAIN) trials, particularly SUSTAIN-6, observed significant reductions in systolic BP with semaglutide, independent of weight loss [2]. Further studies demonstrated that GLP-1 RAs may improve endothelial function, reduce arterial stiffness, and inhibit the renin-angiotensin-aldosterone system (RAAS) [3,4]. This multifactorial effect supports their antihypertensive potential. In vitro and in vivo research highlights GLP-1’s direct vasodilatory effect mediated through nitric oxide (NO) pathways and natriuretic action via the kidney [5]. GLP-1 receptor activation in vascular smooth muscle cells can increase cyclic guanosine monophosphate (cGMP), promoting relaxation and vasodilation [6]. Moreover, the RAAS-inhibitory property of GLP-1 RAs contributes to reduced systemic vascular resistance and improved renal hemodynamics [7]. A meta-analysis by Kristensen et al. in 2019 involving over 56000 participants confirmed a mean systolic BP reduction of 2.6 mmHg and a diastolic reduction of 1.3 mmHg with GLP-1 RAs, supporting their antihypertensive role [8]. Importantly, these effects were more pronounced in patients with higher BMI, reinforcing weight loss as a central mechanism. However, evidence remains limited regarding GLP-1 RAs’ impact on dyslipidemia, especially in patients without baseline lipid abnormalities. Here, presenting a case of an obese diabetic male with uncontrolled hypertension who experienced BP normalization and stable lipid profile over 18 months following semaglutide initiation, with complete withdrawal of oral diabetic medications in 18months. This case illustrates the multifaceted cardiovascular benefits of GLP-1 receptor agonists.

## Case presentation

A 48-year-old male presented to the clinic for evaluation of poorly controlled diabetes and elevated BP. He had a three-year history of T2DM and a one-year history of hypertension. His diabetic management included a combination of oral hypoglycemic agents: tablet glimepiride 2 mg twice daily, tablet metformin 1000 mg twice daily, and tablet pioglitazone 30 mg once daily for three years. For hypertension, he had previously been prescribed a triple combination of tablet valsartan 160 mg, amlodipine 10 mg, and hydrochlorothiazide 12.5 mg once daily. However, he had discontinued all antihypertensive medications six months prior due to side effects, including dizziness and perceived fatigue. He denied any history of dyslipidemia and was not on lipid-lowering therapy. There was no significant family history of cardiovascular or renal disease.

**Figure 1:**
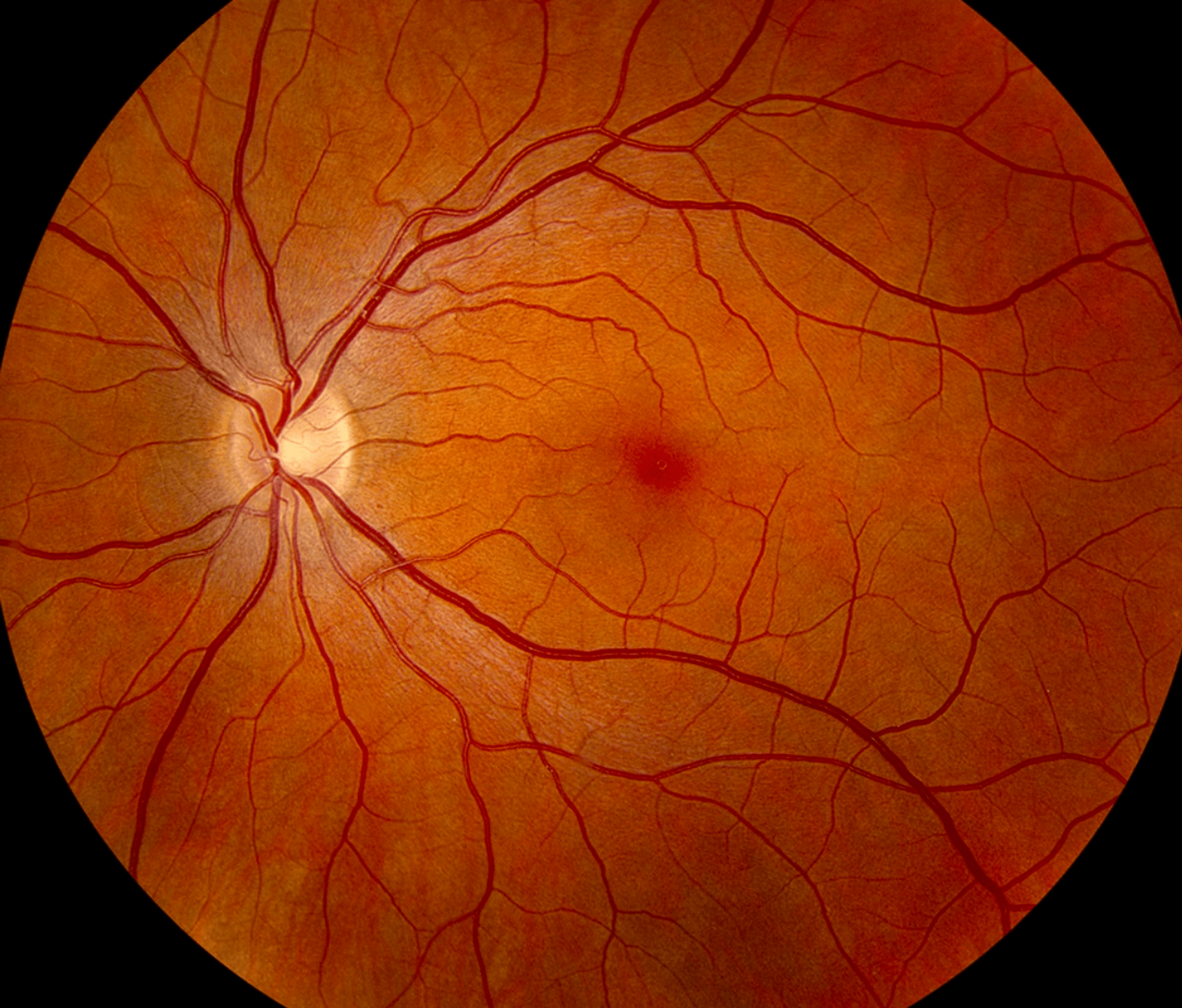
Fundus examination shows normal findings

On examination, he was obese, with a BMI of 38 kg/m². Vital signs revealed a pulse rate of 70/min (regular), a respiratory rate of 18/min, and an average BP of 146/86 mmHg from three readings, measured in the right arm in a sitting position after 15 minutes of rest. No postural hypotension. Systemic clinical examination was normal. Fundus examination (Figure 1) was normal.

Baseline laboratory investigations revealed poor glycemic control with a glycosylated hemoglobin (HbA1c) of 9.2%. Interestingly, the lipid profile was within normal limits: total cholesterol, 178 mg/dL; low-density lipoprotein (LDL-C), 98 mg/dL; high-density lipoprotein (HDL-C), 44 mg/dL; and triglycerides, 140 mg/dL. Lipoprotein(a) was 18 mg/dL, also within the acceptable range. Renal function was normal, with a creatinine level of 0.9 mg/dL, an estimated glomerular filtration rate (eGFR) of 92 mL/min/1.73 m², and a urine albumin-creatinine ratio (UACR) of 18 mg/g, indicating the absence of albuminuria (Table 1).

**Table 1:** Baseline laboratory parameters HbA1c: glycosylated hemoglobin; LDL: low-density lipoprotein; HDL: high-density lipoprotein; ALT: alanine transaminase; eGFR: glomerular filtration rate; UACR: urine albumin-creatinine ratio

Parameter	Result	Reference range
HbA1c	9.2%	<5.7%
Total cholesterol	178 mg/dL	<200 mg/dL
LDL-C	98 mg/dL	<100 mg/dL
HDL-C	44 mg/dL	>40 mg/dL
Triglycerides	140 mg/dL	<150 mg/dL
Lipoprotein(a)	18 mg/dL	<75 mg/dL
Creatinine	0.9 mg/dL	<1.2 mg/dL
ALT	34 mg/dL	<40 mg/dL
eGFR	92 mL/min/1.73m²	>90 mL/min/1.73m²
UACR	18 mg/g	<30 mg/g

He was advised to comply with a strict dietary and exercise regimen. Considering his obesity, poor glycemic control, and the added benefit of weight reduction, a GLP-1 receptor agonist (semaglutide) was initiated at a dose of 0.25 mg once weekly, with plans for gradual titration based on tolerance and glycemic response. Glimepiride was reduced to 1 mg twice daily, pioglitazone was stopped, and metformin continued as before. Patient was not willing for antihypertensives to be restarted at this time because of the past undesirable effects of dizziness and fatigue.

Over the next three months (Table 2), the patient reported good tolerability to semaglutide. He lost 6 kg in weight and his HbA1c improved to 8.0%. His BP had also improved, measuring 134/86 mmHg at the three-month follow-up. The dose of semaglutide was increased to 0.5 mg weekly, and his diabetic medications were continued.

**Table 2:** Follow-up laboratory data over the next 18 months HbA1c: glycosylated hemoglobin; BP: blood pressure

Time point	HbA1c (%)	BP (mmHg)	Weight (kg)	Semaglutide dose
Baseline	9.2	156/96	106	0.25 mg
3 months	8.0	134/86	100	0.5 mg
6 months	7.1	130/82	96	1 mg
9 months	6.6	124/82	92	1 mg
12 months	6.2	122/80	89	0.5 mg
15 months	6.1	120/78	88	0.5 mg
18 months	6.0	118/76	87	0.25 mg

At six months, he had lost a total of 10 kg (weight 96 kg), and his HbA1c further dropped to 7.1%. BP readings were consistently in the range of 130/82 mmHg. Semaglutide was titrated to 1 mg once weekly, glimepiride was reduced to 1 mg once daily in the morning, and metformin was reduced to 750 mg twice daily. Given the favorable trajectory in BP, initiation of antihypertensives was deferred.

By the nine-month mark, semaglutide was continued at 1 mg weekly. The patient had achieved an HbA1c of 6.6%, and BP remained stable at 124/82 mmHg. Lipid profile also showed minor improvements: LDL-C reduced to 88 mg/dL, HDL-C increased to 48 mg/dL, and triglycerides decreased to 118 mg/dL, all without any lipid-lowering therapy. Glimepiride was reduced to 0.5 mg once daily, and metformin was reduced to 500 mg twice daily.

At the 12-month review (Table 2), the patient weighed 89 kg, reflecting a total weight loss of 17 kg from baseline. HbA1c was 6.2%, and BP had normalized to 122/80 mmHg without any pharmacological antihypertensive support. Semaglutide was reduced to 0.5 mg once weekly, as HbA1c was below 6.5%. Glimepiride was discontinued, and metformin was reduced to once daily. Lipid parameters remained within the normal range.

At 15 months, his BP was 120/78 mmHg, and HbA1c was 6.1. His metformin was stopped, and semaglutide was continued at 0.5 mg once weekly. All oral diabetic medications were stopped. At 18 months, BP remained stable at 118/76 mmHg. HbA1c was 6.0% at the final visit. Semaglutide was reduced to 0.25 mg once weekly. He remained asymptomatic, with no hypoglycemia, dizziness, or orthostatic complaints. His Lipid profile showed T cholesterol 150 mg/dL, trilglyceride was 109 mg/dL, LDL was 73 mg/dL, and HDL was 52 mg/dL.

**Figure 2:**
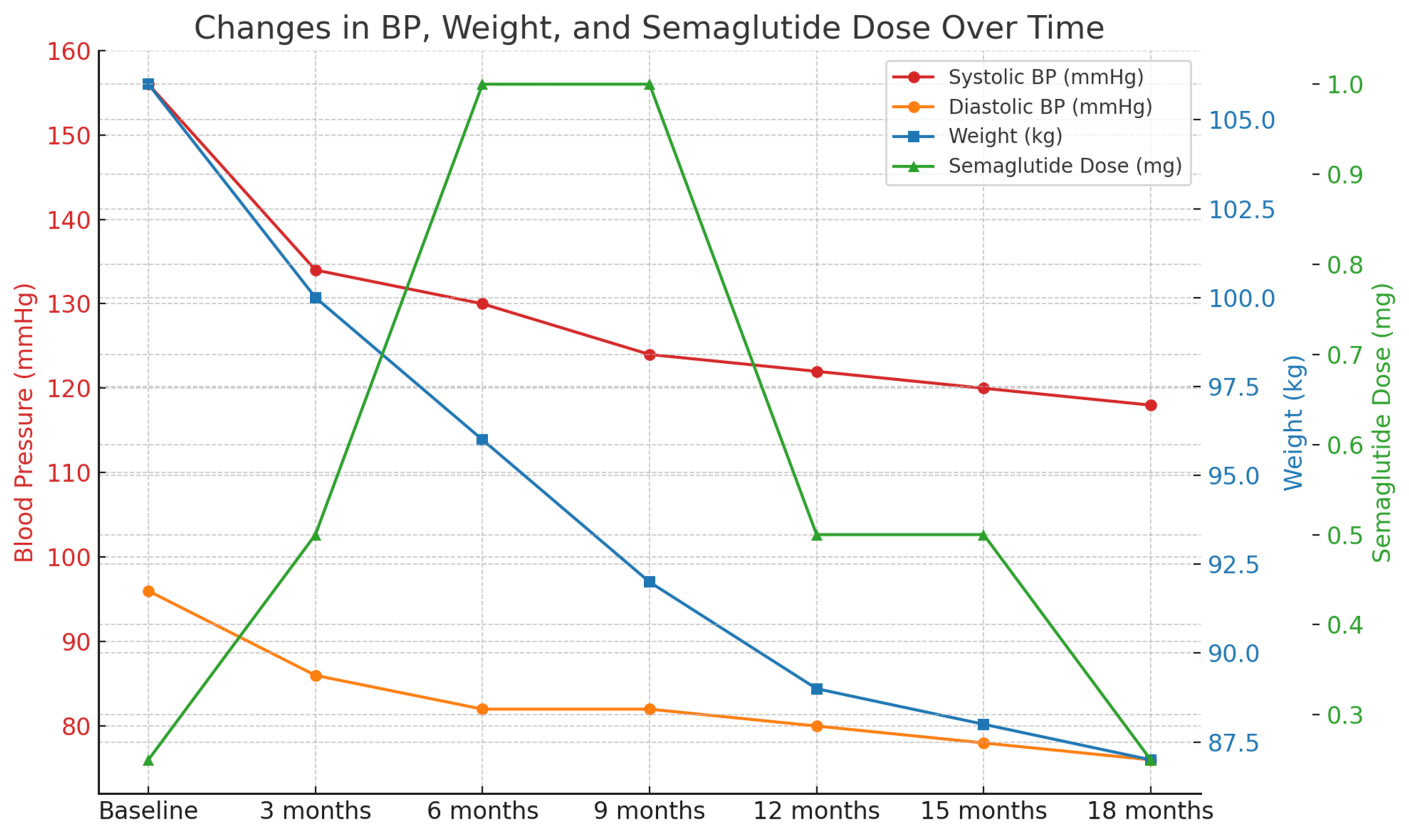
Changes in HbA1c, weight, and BP over 18 months with semaglutide HbA1c: glycosylated hemoglobin; BP: blood pressure

Throughout the 18-month follow-up period (Figure 2), the patient did not receive any antihypertensive or lipid-lowering medications. His consistent BP control, glycemic improvement, and modest lipid benefits can be attributed to the metabolic effects of semaglutide, in addition to the change in diet and exercise regimen and associated weight loss. No adverse effects were reported, and the patient expressed high satisfaction with the treatment regimen.

This case demonstrates that semaglutide, in addition to its glycemic efficacy, can lead to meaningful reductions in BP and improvements in lipid parameters, even in the absence of concurrent cardiovascular medications. The ability to not initiate antihypertensives without loss of BP control highlights the broader systemic benefits of GLP-1 receptor agonist therapy in high-risk metabolic patients. Regular monitoring, patient engagement, and dietary and lifestyle reinforcement were crucial to achieving these outcomes.

## Discussion

This case underscores the multidimensional benefit of GLP-1 RA therapy, particularly semaglutide, in achieving glycemic, BP, and weight control. Despite discontinuing antihypertensive medications at the six-month mark, the patient maintained stable BP over 18 months, suggesting a sustained antihypertensive effect of semaglutide. GLP-1 RAs are not traditionally prescribed for hypertension but consistently demonstrate BP-lowering effects in trials like SUSTAIN-6 [2]. While weight loss contributes significantly to BP reduction, our patient achieved BP control even after plateauing weight loss, indicating alternative mechanisms. Direct vasodilatory effects via GLP-1 receptors on vascular smooth muscle and endothelial cells may be key [6]. Stimulation of cGMP and NO production leads to smooth muscle relaxation, reducing vascular resistance [5]. Semaglutide may also reduce sympathetic tone and exert natriuretic effects by increasing renal sodium excretion [7]. The role of RAAS inhibition is especially relevant in hypertensive diabetic individuals, as seen in animal models and human studies [4]. This case also highlights semaglutide’s feasibility as a single-agent strategy in select high-risk patients. In individuals with obesity, T2DM, and hypertension, the ability to simplify regimens while achieving broad metabolic and cardiovascular control is clinically valuable. GLP-1 RAs also show promise in non-diabetic populations. Evidence suggests that the BP-lowering effect might extend to non-diabetics, particularly if weight loss is a central mechanism. Other proposed mechanisms include GLP-1 receptor activation in the vascular and renal systems, promoting vasodilation via NO and cGMP pathways, improving endothelial function, promoting natriuresis, and inhibiting the RAAS. [5-7]. More comprehensive trials are needed to delineate these mechanisms and to investigate long-term outcomes in patients without diabetes. Nonetheless, the sustained benefit seen in this patient supports broader use of semaglutide, especially in settings where polypharmacy poses challenges.

## Conclusions

This case highlights the multifaceted benefits of semaglutide in a patient with type 2 diabetes, obesity, and uncontrolled hypertension. Alongside improved glycemic control, the patient achieved significant weight loss and sustained BP reduction, allowing the non-initiation of multiple antihypertensive medications. Notably, these improvements suggest broader cardiometabolic effects of GLP-1 agonists. Semaglutide may be a valuable option for patients with overlapping features of diabetes and hypertension, particularly those intolerant to polypharmacy or experiencing therapeutic inertia. Further randomized studies are warranted to define their role in non-diabetic hypertensives and to explore long-term cardiovascular outcomes in broader populations.
